# OA08 Ocrelizumab in pregnancy in a patient with juvenile onset systemic lupus erythematosus

**DOI:** 10.1093/rap/rkae117.008

**Published:** 2024-11-05

**Authors:** Yasir Suleman-Raza, Debajit Sen, Corinne Fisher, Maria Mouyis

**Affiliations:** Rheumatology, Luton and Dunstable University Hospital, Luton, United Kingdom; Department of Adolescent Rheumatology, University College London Hospitals NHS Foundation Trust, London, United Kingdom; Department of Adolescent Rheumatology, University College London Hospitals NHS Foundation Trust, London, United Kingdom; Rheumatology, Luton and Dunstable University Hospital, Luton, United Kingdom

## Abstract

**Introduction:**

Juvenile-onset systemic lupus erythematosus (jSLE) is a multisystem autoimmune disease that results in increased disease activity compared to adult-onset SLE. jSLE in pregnancy results in a greater incidence of disease flares as well as adverse pregnancy outcomes. Management of SLE in pregnancy is particularly difficult due to lack of safety data for immunosuppressant medications and is therefore limited. Ocrelizumab is a humanised monoclonal antibody that selectively targets CD20 B cells and is approved for the treatment of multiple sclerosis. Despite never being trialled in a patient with active SLE in pregnancy, this case demonstrates its use with a successful outcome.

**Case description:**

An 18-year-old female was diagnosed at age 14 with juvenile onset systemic lupus erythematosus with the presence of a photosensitive malar rash, arthralgia, alopecia, aphthous ulcers and headache. At the time of diagnosis her investigations revealed ANA 1:5120 with homogenous pattern, high dsDNA, low C3 and C4, thrombocytopenia, antibodies to Smith, Ro, RNP, anti-Rib-P, strongly positive lupus anticoagulant antibodies and low titre anti-cardiolipin IgG and IgM. Previous treatment included methotrexate, mycophenolate mofetil and cyclosporin which were discontinued either due to patient choice, inefficacy or adverse reaction. Azathioprine was contraindicated due to low TMPT. Rituximab was trialled and although effective had to be stopped due to adverse reaction. Unfortunately, she experienced a primigravida pregnancy loss at 15-20 weeks secondary to active SLE and pre-eclampsia. Shortly after this, she developed a rare case of anti-fascin antibody neuropathy and was treated with ocrelizumab. Six months later during her second pregnancy, she was treated with hydroxychloroquine 400 mg, aspirin 150 mg, folic acid 5 mg and low molecular weight heparin prophylaxis which were continued throughout the pregnancy. Due to concerns of active lupus at 13 weeks due to facial rash and fatigue, prednisolone was commenced at 10 mg daily and continued. After careful consideration, Ocrelizumab was given in the second trimester at 17 and 19 weeks’ gestation. Following this, symptoms remained under control and disease activity was low with normal C3/C4 and dsDNA levels without significant proteinuria. A newborn baby was delivered at 38 weeks by caesarean section without complication or foetal abnormalities. Both the mother and baby remain under close follow- up but have not demonstrated adverse outcomes at 3 months postpartum.

**Discussion:**

This case demonstrates the use of Ocrelizumab in the second trimester of a high-risk pregnancy complicated by active jSLE. At present, there is no license for use of Ocrelizumab in SLE and no safety data on its use in pregnancy. However, there was a good outcome for this patient which resulted in no antenatal or postpartum complications and the successful birth of a healthy newborn without foetal abnormalities.

Ocrelizumab is used for the treatment of early relapsing-remitting multiple sclerosis (RRMS) and is increasingly used by neurologists in women of childbearing age despite being unlicensed for use in pregnancy. Concerns of its use in pregnancy surround B cell depletion in the neonate which while tends to be transient, causes significant concern regarding long term immunological development and response to vaccination. B cell depleting therapies are widely used in the context of active and refractory SLE and therefore use of Ocrelizumab has been evaluated in cases of SLE in two phase III clinical trials. The first study examined the efficacy of Ocrelizumab in SLE without renal involvement (BEGIN) and the second recruited patients with lupus nephritis (BELONG). The BEGIN study was terminated early due to lack of response and the BELONG study was terminated due to a significant increase in serious infections (in the Ocrelizumab plus mycophenolate treatment group). However, results on disease activity were positive with complete renal response being achieved non-statistically more frequently in the Ocrelizumab treated patients versus placebo.

Real world evidence shows the importance of achieving disease control in SLE around pregnancy to prevent adverse outcomes. The decision for the use of Ocrelizumab therefore needs to be balanced carefully against potential harm to the baby either from direct foetal exposure to the drug, or from gestation in an immunocompromised mother.

**Key learning points:**

• Pre-pregnancy counselling is of paramount importance in severe cases of active SLE and when possible, pregnancy should be postponed in these instances until disease activity is stabilised with contraception counselling offered to those on teratogenic immunosuppressive agents.

• A multidisciplinary approach to the management of complex SLE in pregnancy in essential with obstetrician, midwife, maternal medicine physician and rheumatologist input.

• The benefit of potentially life-saving immunosuppressive treatment should be carefully balanced against the poor outcomes of active SLE in pregnancy and decisions should be made on a case-by-case basis.

